# Differences in P-glycoprotein activity in human and rodent blood–brain barrier assessed by mechanistic modelling

**DOI:** 10.1007/s00204-021-03115-y

**Published:** 2021-07-15

**Authors:** Laurens F. M. Verscheijden, Jan B. Koenderink, Saskia N. de Wildt, Frans G. M. Russel

**Affiliations:** 1 Department of Pharmacology and Toxicology, Radboud Institute for Molecular Life Sciences, Radboud University Medical Center, Nijmegen, The Netherlands; 2 Intensive Care and Department of Paediatric Surgery, Erasmus MC-Sophia Childrens Hospital, Rotterdam, The Netherlands

**Keywords:** Physiologically based pharmacokinetic modelling, P-glycoprotein, Species differences, Blood–brain barrier, Paediatric, Brain

## Abstract

Variation in the efficacy and safety of central nervous system drugs between humans and rodents can be explained by physiological differences between species. An important factor could be P-glycoprotein (Pgp) activity in the blood–brain barrier (BBB), as BBB expression of this drug efflux transporter is reportedly lower in humans compared to mouse and rat and subject to an age-dependent increase. This might complicate animal to human extrapolation of brain drug disposition and toxicity, especially in children. In this study, the potential species-specific effect of BBB Pgp activity on brain drug exposure was investigated. An age-dependent brain PBPK model was used to predict cerebrospinal fluid and brain mass concentrations of Pgp substrate drugs. For digoxin, verapamil and quinidine, in vitro kinetic data on their transport by Pgp were derived from literature and used to scale to in vivo parameters. In addition, age-specific digoxin transport was simulated for children with a postnatal age between 25 and 81 days. BBB Pgp activity in the model was optimized using measured CSF data for the Pgp substrates ivermectin, indinavir, vincristine, docetaxel, paclitaxel, olanzapine and citalopram, as no useful in vitro data were available. Inclusion of Pgp activity in the model resulted in optimized predictions of their brain concentration. Total brain-to-plasma AUC values (Kp,brain) in the simulations without Pgp were divided by the Kp,brain values with Pgp. Kp ratios ranged from 1 to 45 for the substrates investigated. Comparison of human with rodent Kp,brain ratios indicated ≥ twofold lower values in human for digoxin, verapamil, indinavir, paclitaxel and citalopram and ≥ twofold higher values for vincristine. In conclusion, BBB Pgp activity appears species-specific. An age-dependent PBPK model-based approach could be useful to extrapolate animal data to human adult and paediatric predictions by taking into account species-specific and developmental BBB Pgp expression.

**Supplementary Information:**

The online version contains supplementary material available at 10.1007/s00204-021-03115-y.

## Introduction

Rodent studies are performed during non-clinical development of drug candidates for the assessment of their effectivity and safety. Detection of central nervous system toxicity is not always sensitive enough, as it remains one of the main causes for discontinuation due to safety reasons in the clinical phase of drug development (Weaver and Valentin 2019). In chemical risk assessment, potentially neurotoxic agents are tested in rodents, but findings cannot always be extrapolated to humans (Krewski et al. 2010). Species differences between rodents and humans are the reason for the lack of predictive value and can result from both altered characteristics in pharmacokinetics as well as mode of action (Jones et al. 2013).

Blood–brain barrier (BBB) function is an important factor mediating neurotoxicity. The efflux transporter P-glycoprotein (Pgp) is a major player in restricting brain access to xenobiotics, as indicated by mice knockout studies and human drug–drug interaction studies (Bauer et al. 2015; Morris et al. 2017; Xie et al. 1999). Species differences in Pgp expression and activity could result in an inaccurate estimate of xenobiotic BBB penetration and neurotoxic potential. Previous studies suggested that Pgp expression is lower in humans compared to rodents (Al Feteisi et al. 2018; Uchida et al. 2011). In addition, prediction of Pgp activity is complicated by differences in expression between subgroups, as, for instance, Pgp expression in children is lower compared to adults, potentially making this population more susceptible to higher substrate brain exposures (Lam et al. 2015; Verscheijden et al. 2020b).

In vitro-to-in vivo extrapolation (IVIVE) is used to scale in vitro kinetics to parameters that reflect transport in vivo. In vitro transporter activity is corrected for the amount of transporter protein in the in vitro system and multiplied with abundance in the tissue (*e.g.* BBB) of interest (Bhatt et al. 2019; Cheung et al. 2019). This allows predictions of BBB Pgp activity for the human transporter isoform. In addition, predictions for sub-groups such as children are possible by correcting for age-related differences in transporter expression (Verscheijden et al. 2020a). This has previously been used for predicting liver and kidney exposure, and recent studies proposed a similar approach for the brain (Kumar et al. 2018; Li et al. 2017; Neuhoff et al. 2013a; Verscheijden et al. 2021).

Over the last decades, IVIVE in combination with physiologically based toxicokinetic/pharmacokinetic modelling (PBTK/PBPK) has become a versatile tool for first-in-human dose selection, prediction of DDI’s, PK in special populations and prediction of internal organ exposure (Paini et al. 2019; Rose et al. 2014; Shebley et al. 2018; Verscheijden et al. 2020a). An advantage of PBPK modelling is that predictions are based on system-specific properties (also called physiological properties) which are, as much as possible, separated from drug-related properties. This allows the re-use of models for different compounds by changing the drug-related parameters, or extrapolation of models to other populations/species by changing physiological parameters. By this means, models have been extrapolated from animals to humans and from adults to children (Verscheijden et al. 2020a). In addition, due to the multi-compartment structure, models have been used for the prediction of tissue drug concentrations. Including IVIVE parameters in a PBPK model allows for the prediction of brain drug concentrations (Gaohua et al. 2016) and the influence of BBB Pgp activity can be quantified (Li et al. 2017).

In this study, a human PBPK model was used that included blood–brain barrier Pgp activity. Predictions of adult and paediatric brain exposures with and without the inclusion of Pgp were compared with reported brain exposure in rodent wildtype, knockout and Pgp-inhibited animals. By this means, the potential effect of species-specific Pgp-mediated BBB function on differences in observed central nervous system drug exposure was assessed.

## Methods

### General human brain PBPK model development

**Fig. 1:**
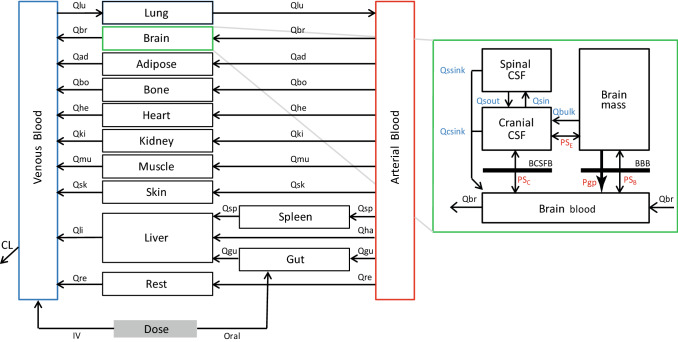
Schematic outline of the PBPK model including four brain compartments (adapted from Verscheijden et al. (Verscheijden et al. 2019)). Qsin and Qsout represent CSF shuttle flow between cranial CSF and spinal CSF compartments. Qssink and Qcsink are the flows from CSF compartments to blood. Qbulk represents bulk flow from brain mass to cranial CSF. PS_B_, PS_C_ and PS_E_ represent permeability surface area products between brain blood and brain mass, brain blood and cranial CSF, and brain mass and cranial CSF, respectively. Pgp represents active BBB Pgp-mediated transport. Subscripts lu, br, ad, bo, he, ki, mu, sk, li, re, gu, sp, ha denote lung, brain, adipose tissue, bone, heart, kidney, muscle, skin, liver, rest tissue, gut, spleen and hepatic artery, respectively. CL is the total clearance from the model. IV and oral indicate intravenous and oral route of administration

A 14 compartment PBPK model was developed in Rstudio version 3.6.2 based on a model published previously (Gaohua et al. 2016; Verscheijden et al. 2019, 2021) (Fig. 1). Physiological parameters for body weight, body height, body surface area, organ volumes, tissue blood flows, haematocrit and albumin concentrations were included. Organ-plasma partitioning was estimated using the equations of Rodgers and Rowland, which take into account tissue composition, ionization and lipophilicity of compounds (Online Resource 1) (Rodgers et al. 2005; Rodgers and Rowland 2006). Drug clearance, Kp values and oral absorption rate constants were derived from clinical studies and optimized to capture measured plasma data if necessary (Online Resource 1). The clearance coefficient of variation was set to 30% for all compounds. Specifically for simulations in children, age-appropriate physiological parameters were included as reported previously (Verscheijden et al. 2019).

The part of the model describing the brain consists of four compartments, representing brain mass, intracranial CSF, spinal CSF, and brain blood (Gaohua et al. 2016; Verscheijden et al. 2019). In short, physiological parameters were included for organ volumes, blood and CSF fluid flows, and BBB and BCSFB surface area (Verscheijden et al. 2019). The BBB is the barrier between brain mass and blood. The blood–CSF barrier is the barrier between the cranial CSF and blood and is assumed to be half of the BBB surface area (Verscheijden et al. 2019). No barrier exists between brain mass and cranial CSF, and between spinal and cranial CSF, which are connected via CSF shuttle flow (Gaohua et al. 2016). All compartments were treated as well stirred. For simulations in children, age-appropriate brain physiological parameters were included as reported previously (Verscheijden et al. 2019).

### Parameters for substrates entering the brain by passive diffusion: *quetiapine, oxycodone, mirtazapine, etoricoxib, dexketoprofen, lacosamide and ibuprofen*

To confirm that the physiological parameters used in the model are accurate, simulations were performed for quetiapine, oxycodone, mirtazapine, etoricoxib, dexketoprofen, lacosamide and ibuprofen assumed to enter the brain via passive diffusion (Boström et al. 2005; Moons et al. 2011; O'Brien et al. 2013; Schmitt et al. 2012; Uhr et al. 2003). The BBB and BCSFB permeability surface area product was calculated using apparent permeability (Papp) values from in vitro assays in MDCK or Caco-2 cell lines, as follows (Li et al. 2017):

1 $$PSbbb = Papp,invitro \times BBB\;surface\;area$$

where PSbbb is the permeability surface area product of the BBB. Papp, in vitro is the Papp in dm/h and BBB surface area is the surface area of the blood–brain barrier in dm^2^. Multiple Papp values were averaged, if available. Binding of drugs to brain components was reflected by the unbound fraction in brain mass (fubm) derived from animal experiments, which show good correlation with human values, or in silico predicted fractions (Online Resource 1). Only unbound drug is assumed to cross the brain barriers (Verscheijden et al. 2019).

### Parameters for Pgp substrates with in vitro transport data: *digoxin, quinidine and verapamil*

For Pgp substrates digoxin, verapamil, quinidine (Bauer et al. 2012; Kusuhara et al. 1997; Mayer et al. 1997; Pussard et al. 2007; Römermann et al. 2013; Sadiq et al. 2015; Schinkel et al. 1995), in vitro Caco-2 maximum rate of transport (Vmax) and affinity constant (Km) parameters were extracted from literature, in addition to passive permeability (Papp) and brain binding (fubm) parameters (Online Resource 1). Digoxin and verapamil efflux parameters have been incorporated previously in liver and intestinal PBPK model compartments (Neuhoff et al. 2013a, b). Vmax values were corrected for differences in Pgp protein abundance between the in vitro cell system and in vivo blood–brain barrier micro-vessels and scaled to an in vivo parameter using the equation:

2 $${V_{\max bbb}} =\; {V_{\max ,vitro}}/\Pr ocell * \frac{{Pgp\;abundance(Mv)}}{{Pgp\;abundance(Caco2)}} * BMvPGB * BW$$

where Vmaxbbb is the maximum rate of transport in the total BBB in pmol/min and Vmax,vitro is the maximum rate of transport in Caco-2 cells in pmol/min/cm2. Procell is the amount of protein in the in vitro system, which was assumed to be 150 ug/cm2. Pgp abundance(Caco2) represents the Pgp protein abundance in the intestinal-derived Caco-2 cell-line and Pgp abundance(Mv) the Pgp protein abundance in the endothelial BBB microvessels, which was reportedly on average 0.9 and 4.21 pmol/mg total protein, respectively (Al-Majdoub et al. 2019; Brück et al. 2017; Shawahna et al. 2011; Uchida et al. 2011). BMvPGB, the amount of brain micro-vessel per gram of brain, was reported to be 244 ug protein/g brain, and the value used for BW (brain weight) was age-dependent (as reported previously (Verscheijden et al. 2019)), and assumed to be 1400 g in adults (Li et al. 2017).

Pgp-mediated transport was incorporated in time-based differential equations describing the rate of change in brain and blood drug concentration according to the following Michaelis Menten equation:

3 $$\frac{{dA,active}}{{dt}} = \frac{{f\exp * V\max ,bbb * fubm * Cbm}}{{Km + fubm * Cbm}}$$

where fubm is the fraction of unbound drug in brain mass and Cbm the total concentration of drug in brain mass. Active efflux transport results in a reduction in the amount of drug present in the brain compartment, while it will increase the amount of drug in the blood compartment over time (dA,active/dt). For simulations in the paediatric population, Vmax was multiplied with the relative Pgp expression (fexp = 0.57) compared to adult expression reported by Lam et al. (Lam et al. 2015).

### Parameters for Pgp substrates without in vitro transport data: *ivermectin, indinavir, vincristine, docetaxel, paclitaxel, olanzapine and citalopram*

Simulations were also performed for Pgp substrates for which no in vitro transporter activity was available (Bundgaard et al. 2012; Chu et al. 2012; Gallo et al. 2003; Geyer et al. 2009; Kemper et al. 2003; Kiki-Mvouaka et al. 2010; Kim et al. 1998; Schinkel et al. 1994; Uhr and Grauer 2003; Wang et al. 2010, 2004). In these models, Pgp activity was optimized to predict measured CSF data (reported in “Model verification and parameter optimization using published clinical studies” below) using the equation:

4 $$\frac{{dA,active}}{{dt}} = CLpgp * fubm * Cbm$$

where CLpgp is the (optimized) BBB-mediated Pgp efflux clearance in L/h, fubm is the unbound fraction of drug in brain, and Cbm is the total brain drug concentration. The optimized Pgp clearance parameters and other drug-related parameters are reported in Online Resource 1.

### Model verification and parameter optimization using published clinical studies

For model verification (digoxin, verapamil and quinidine) and optimization of Pgp efflux clearance (ivermectin, indinavir, vincristine, docetaxel, paclitaxel, olanzapine, and citalopram), clinical studies with relevant plasma and CSF drug concentrations were used. Studies included patients suffering from a wide variety of conditions or receiving co-medication, which potentially could have affected drug brain disposition. A summary of all clinical studies and their characteristics is reported in Table 1. Only for digoxin, a study in children was used.

**Table 1:** Characteristics of studies used for model verification and parameter optimization

Drug	Number of patients	Dose	Co-medication	Age	Indication	CSF PK sample collection
No transporter substrates						
Quetiapine (Nikisch et al. 2010)	22	600 mg/day oral	–	18–55 y	Schizophrenic episode	Lumbar puncture
Oxycodone (Kokki et al. 2014)	11	0.092 mg/kg IV	Diazepam, paracetamol, midazolam, propofol, remifentanil, rocuronium, sevoflurane	26–60 y	Postoperative epidural analgesia	Epidural catheter
Mirtazapine (Paulzen et al. 2015)	16	33.3 mg/day oral	Quetiapine, venlafaxine, citalopram	28–78 y	Major depressive episode	Lumbar puncture
Etoricoxib (Piirainen et al. 2016)	12	60 mg oral	Paracetamol, levobupivacaine, fentanyl, oxycodone	56–72 y	Total hip arthroplasty	Spinal catheter
Dexketoprofen (Piirainen et al. 2016)	12	0.5 mg/kg IV	Paracetamol, levobupivacaine, fentanyl, oxycodone	53–71 y	Total hip arthroplasty	Spinal catheter
Lacosamide (May et al. 2015)	21	166 mg/12 h oral	Various anti-epileptic drugs	18–65 y	Epilepsy	Lumbar puncture
Ibuprofen (Brazier et al. 2017)	26	10 or 20 mg oral	Cromolyn	55–75 y	Healthy volunteers	Lumbar puncture
Pgp substrates						
Digoxin (Allonen et al. 1977)	11 adults 8 infants	0.0032 mg/kg/day oral (adult) 0.011 mg/kg/day oral (infant)	Not available	68–92 y (adult) 25–81 d (infant)	Disease not specified (adult) Hearth failure/hydrocephalus (infants)	Lumbar puncture
Verapamil (Narang et al. 1988)	7	480 mg/day oral	–	22–44 y	Schizophrenia	Lumbar puncture
Quinidine (Ochs et al. 1980)	8	385 mg/12 h oral	Not available	23–70 y	Volunteers scheduled for lumbar puncture	Lumbar puncture
Ivermectin (Rose et al. 2009)	1	30 g/day oral	Broad spectrum antibiotics	59 y	Lymphocytic leukaemia, Strongyloides stercoralis infection	Lumbar puncture
Indinavir (Haas et al. 2000)	8	800 mg/8 h oral	Zidovudine, lamivudine, stavudine	31–50 y	HIV infection	Lumbar intrathecal catheter
Vincristine (Jackson et al. 1981)	2	2 mg IV	Methotrexate	60–66 y	Non-Hodgkin’s lymphoma or leukaemia	Ventricular catheter
Docetaxel (ten Tije et al. 2004)	1	75 mg/m2 IV	Not available	Not available	Metastatic breast cancer	Lumbar puncture
Paclitaxel (Chen et al. 2006)	6	175 mg/m2 IV	Dexamethasone, phenytoin	34–73 y	Original brain tumour or brain metastases	Ommaya reservoirs or lumbar puncture
Olanzapine (Skogh et al. 2011)	29	11.6 mg/day oral	Benzodiazepines, zopiclone	23–50 y	Schizophrenia or schizoaffective disorder	Lumbar puncture
Citalopram (Paulzen et al. 2016)	18	21.1 mg/day oral	–	28–84 y	Different psychiatric diagnoses	Lumbar puncture

Simulations were performed using a virtual population of 100 individuals, who were matched with the original clinical study for dosing regimen, age range and fraction female/male, if reported. Simulated median, 5th percentile, 95th percentile, minimum and maximum concentration–time profiles were compared with observed values from clinical studies. In addition, plasma and CSF prediction errors were calculated as performed previously according to the equation (Yamamoto et al. 2017):

5 $$PE = \frac{{Yobs,i - Ypred,median,i}}{{\left( {Yobs,i + Ypred,median,i} \right)/2}}$$

where Yobs,i is the ith individual or mean observation in the clinical study at a specific point in time and Ypred,median,i is the median concentration predicted at the same point in time. Variability in the clinically measured PK values is assumed to cancel out in the analysis. Therefore, median PE ideally equals 0. A median PE of ± 0.667 and ± 1 refer to a twofold or a threefold median difference between predicted and observed values, respectively.

### Assessment of species-specific Pgp activity

Human adult and pediatric simulations were performed with and without Pgp-mediated active transport. Effects of Pgp on brain mass area under the curve (AUC) and plasma AUC were quantified for one dosing interval when the system was at steady state using the (prolonged) dosing regimen described in the clinical studies (Table 1). Clinical studies with vincristine and docetaxel only reported single dose PK data; therefore, dosing was repeated weekly for vincristine or once every three weeks for docetaxel. Total brain-to-plasma AUC ratios (Kp,brain), in the situation where Pgp was not active, were divided by the Kp,brain in case Pgp was considered (i.e. Equations 3 or 4 included in the PBPK model) for adults and children, using the formula:

6 $$Kpratio = \frac{{Kpbrain,Pgpinhbited}}{{Kpbrain,Pgpactive}}$$

The same equation was used to calculate Kp ratios in adult rodent studies for Pgp substrates listed above, where AUC values or single-point drug concentrations in plasma and brain were used to calculate Kp,brain. Kp,brain values in knockout rodents or rodents in which Pgp was inhibited were divided by Kp,brain obtained from wildtype, untreated animals (Bauer et al. 2012; Bundgaard et al. 2012; Chu et al. 2012; Gallo et al. 2003; Geyer et al. 2009; Kemper et al. 2003; Kiki-Mvouaka et al. 2010; Kim et al. 1998; Kusuhara et al. 1997; Mayer et al. 1997; Pussard et al. 2007; Römermann et al. 2013; Sadiq et al. 2015; Schinkel et al. 1994, 1995; Uhr and Grauer 2003; Wang et al. 2010, 2004). Human and rodent Kp ratios were compared and considered different when ratios exceeded an arbitrary value of twofold difference.

## Results

### Substrates entering the brain by passive diffusion

**Fig. 2:**
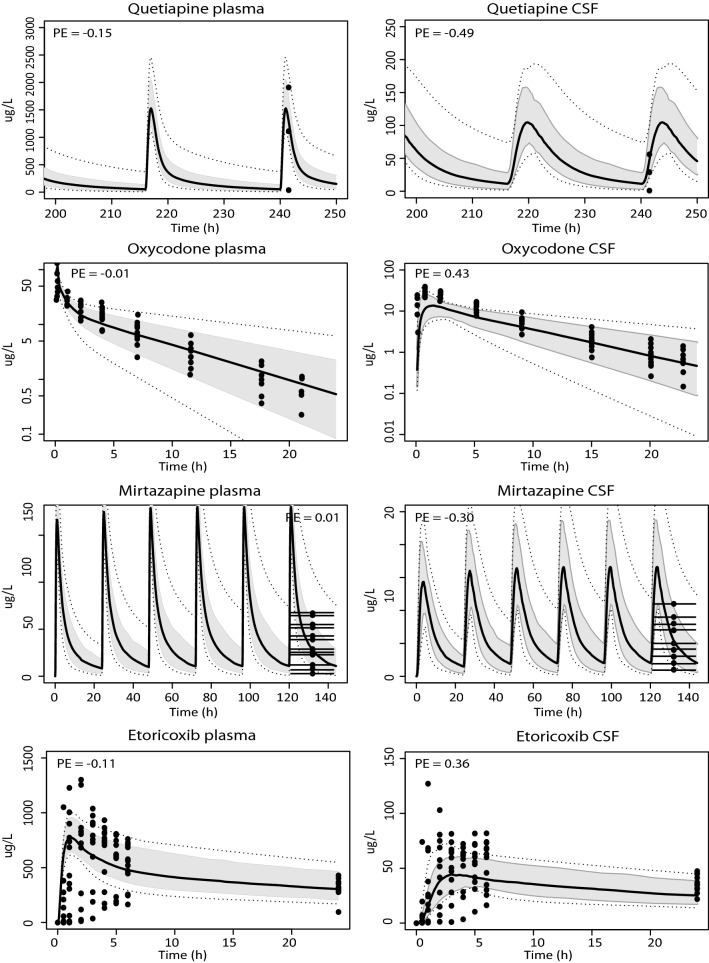

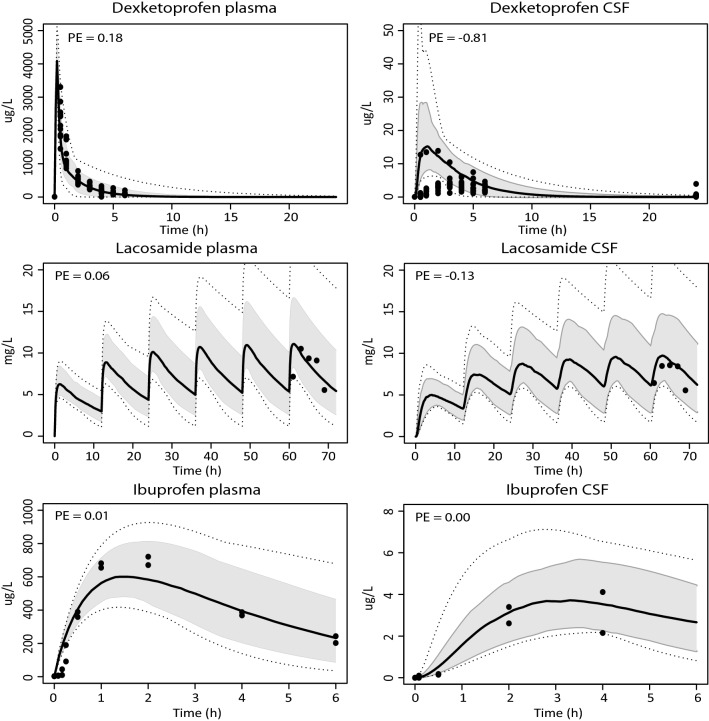
Model simulations for drugs reaching the brain via passive diffusion only. Simulations in plasma and CSF for the compounds quetiapine (600 mg/day, oral), oxycodone (0.092 mg/kg, IV), mirtazapine (33.3 mg/day, oral), etoricoxib (60 mg, oral), dexketoprofen (0.5 mg/kg, IV), lacosamide (166 mg/12 h, oral), and ibuprofen (10 mg, oral). The black solid line indicates the median simulated value. The grey area represents 90% CI in inter-individual variability. Dotted lines indicate minimum and maximum simulated values. Dots are individual or mean observed values. Horizontal lines indicate the range in which measured samples were obtained. Prediction errors where calculated as described in the "Methods" section, ${\text{PE}} = \frac{{{\text{Yobs}},{\text{i}} - {\text{Ypred}},{\text{median}},{\text{i}}}}{{\left( {{\text{Yobs}},{\text{i}} + {\text{Ypred}},{\text{median}},{\text{i}}} \right)/2}}$

The model was first verified with quetiapine, oxycodone, mirtazapine, etoricoxib, dexketoprofen, lacosamide and ibuprofen for which BBB passage is only subject to passive diffusion. Reasonable predictions could be made for most compounds, although the dexketoprofen CSF concentration–time profile was overestimated with a prediction error of −0.81. Overlays of predicted and measured data are shown in Fig. 2 together with calculated prediction errors.

### Pgp substrates with in vitro transport data

**Fig. 3:**
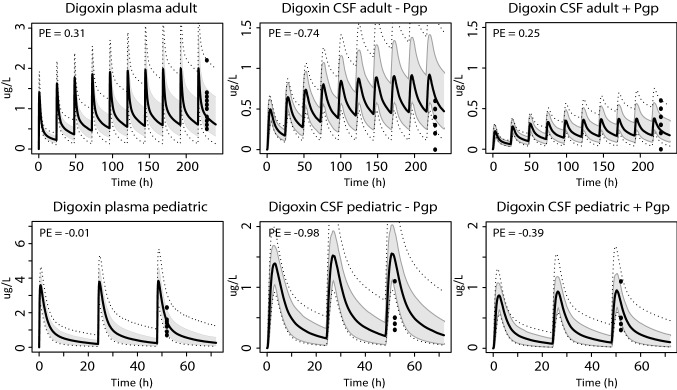
Predictions of digoxin concentrations in plasma and CSF of adults and young children. Simulations of plasma and CSF (with and without Pgp activity) concentrations in adults (0.0032 mg/kg/day digoxin, oral) and young children (0.011 mg/kg/day digoxin, oral). The black solid line indicates the median simulated value. The grey area represents 90% CI in inter-individual variability. Dotted lines indicate minimum and maximum simulated values. Dots are individual observed values. Prediction errors where calculated as described in the "Methods" section, ${\text{PE}} = \frac{{{\text{Yobs}},{\text{i}} - {\text{Ypred}},{\text{median}},{\text{i}}}}{{\left( {{\text{Yobs}},{\text{i}} + {\text{Ypred}},{\text{median}},{\text{i}}} \right)/2}}$

**Fig. 4:**
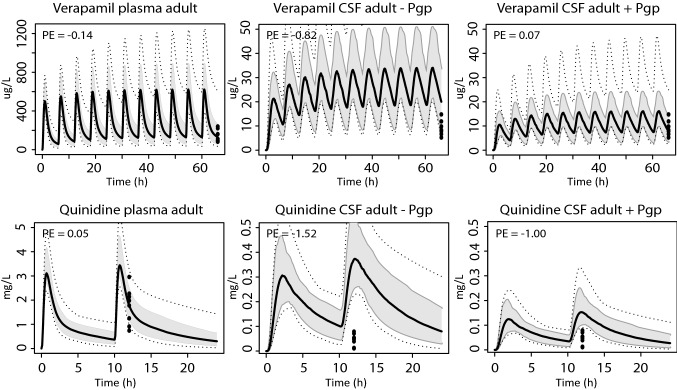
Predictions of verapamil and quinidine concentrations in plasma and CSF of adults. Simulations in plasma and CSF (with and without Pgp activity) after oral doses of 480 mg/day verapamil and 385 mg/12 h quinidine. The black solid line indicates the median simulated value. The grey area represents 90% CI in inter-individual variability. Dotted lines indicate minimum and maximum simulated values. Dots are individual observed values. Prediction errors where calculated as described in the "Methods" section, ${\text{PE}} = \frac{{{\text{Yobs}},{\text{i}} - {\text{Ypred}},{\text{median}},{\text{i}}}}{{\left( {{\text{Yobs}},{\text{i}} + {\text{Ypred}},{\text{median}},{\text{i}}} \right)/2}}$

Pgp transport parameters were included in the model for digoxin, quinidine and verapamil. To optimally capture clinically measured digoxin CSF values, the Pgp protein abundance ratio between Caco-2 and brain micro-vessels in Eq. 2 had to be multiplied with 9. The adjusted value was kept the same for the paediatric digoxin, verapamil and quinidine simulations. Simulated digoxin plasma and CSF PK profiles with and without active Pgp transport are shown in Fig. 3. Inclusion of active Pgp transport resulted in an improved overlay between simulated and measured concentrations in CSF in adults and children between 25 and 81 days postnatal age, as shown by the plots and measured median prediction errors (Fig. 3). In addition, adding Pgp activity to the model resulted in a better agreement between simulated and measured verapamil and quinidine CSF drug concentrations with median prediction errors more closely to 0 (Fig. 4).

### Pgp substrates without in vitro transport data

**Fig. 5:**
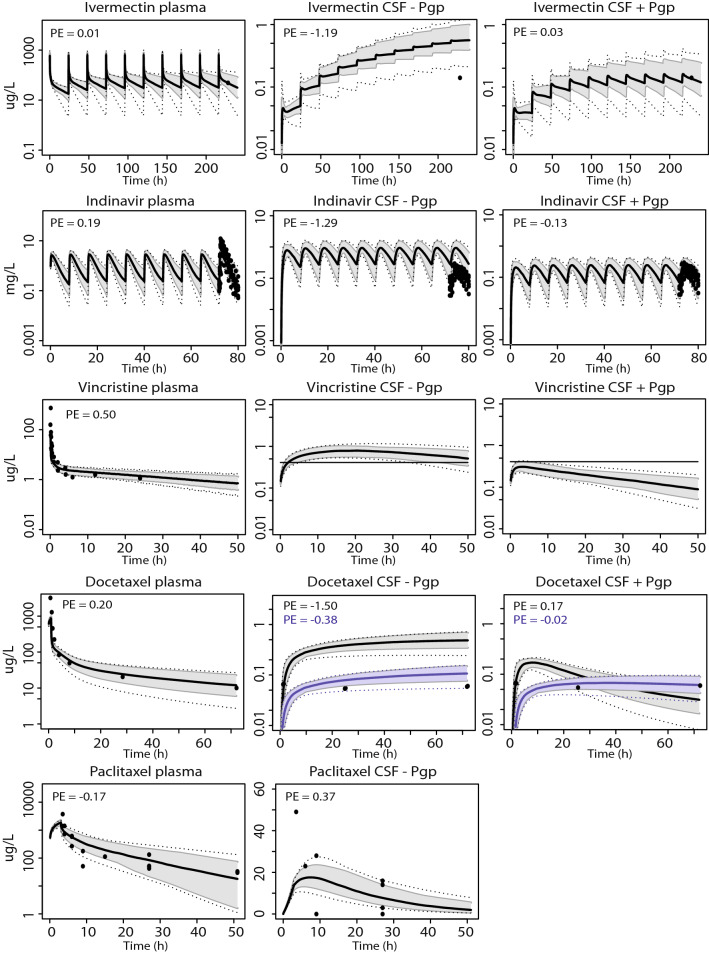

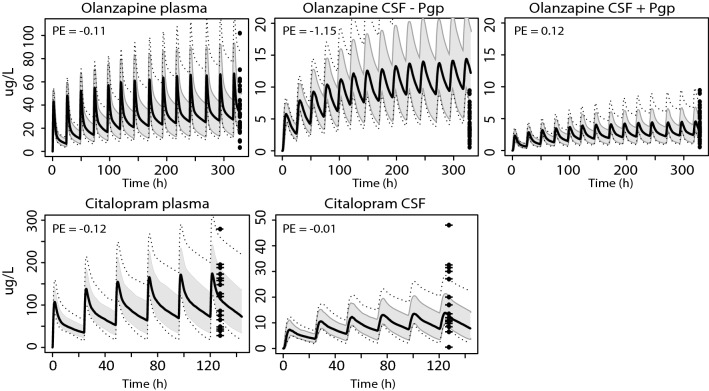
Model simulations for seven additional Pgp substrates Simulations in plasma and CSF (with and without Pgp activity) for the compounds ivermectin (30 g/day, oral), indinavir (800 mg/8 h, oral), vincristine (2 mg, IV), docetaxel (75 mg/m2, IV), paclitaxel (175 mg/m2, IV), olanzapine (11.6 mg/day, oral), and citalopram (21.1 mg/day, oral). The black solid line indicates the median simulated value. The grey area represents 90% CI in inter-individual variability. Dotted lines indicate minimum and maximum simulated values. Dots are individual observed values. The horizontal line indicates the vincristine lower limit of quantification. Blue lines indicate docetaxel simulations using 20% and 12.5% of original passive permeability (PSbbb) and BBB Pgp activity (CLpgp) parameter values, respectively. Prediction errors where calculated as described in the "Methods" section, ${\text{PE}} = \frac{{{\text{Yobs}},{\text{i}} - {\text{Ypred}},{\text{median}},{\text{i}}}}{{\left( {{\text{Yobs}},{\text{i}} + {\text{Ypred}},{\text{median}},{\text{i}}} \right)/2}}$

Model simulations were also performed for the Pgp substrate drugs ivermectin, indinavir, vincristine, docetaxel, paclitaxel, olanzapine and citalopram. Pgp activity was quantified by optimization of the CLpgp parameter in the model with CSF measurements, although this parameter was not needed to obtain an overlay with measured data for paclitaxel and citalopram (Fig. 5). The predicted and observed data did correspond well for all drugs after CLpgp optimization, except for docetaxel, as a relatively constant measured CSF concentration did not match with the simulated profile. A reduction of the BBB and BCSFB passive permeability product improved the model-based prediction for this drug (Fig. 5). Including Pgp activity had little influence on the simulated plasma concentration–time curves for all compounds investigated, with steady-state concentrations being < 1% different from the situation where BBB Pgp was not considered (data not shown).

### Assessment of species-specific Pgp activity

Plasma concentration-corrected brain concentrations (Kp,brain values) were calculated with and without Pgp activity and Kp ratios ranged from 1.0 to 45 (Table 2). Docetaxel was not considered due to the initial mismatch between predicted and observed values. Data reported in rodent knockout and inhibition studies were included, to evaluate the difference with human predicted values (Table 2). Human model-based predictions for digoxin, verapamil, indinavir, paclitaxel and citalopram resulted in ≥ twofold lower Kp ratios compared to rodent studies. In contrast, the Kp ratio of vincristine was ≥ twofold higher in the human model simulation compared to the values found in rodents (Table 2).

**Table 2:** Kp ratios in the human brain PBPK model (with and without Pgp) versus Kp ratios from rodent studies (with and without Pgp)

Compound	Kp ratios human PBPK model	Kp ratios in rat/mouse studies
Digoxin (adult 35y)	2.7	10.0–27.8 (Mayer et al. 1997; Schinkel et al. 1995)
Digoxin (child 1mnd)	2.0	10.0–27.8 (Mayer et al. 1997; Schinkel et al. 1995)
Verapamil	2.3	5.3–30 (Bauer et al. 2012; Römermann et al. 2013; Sadiq et al. 2015)
Quinidine	2.7	4.2–27.6 (Kusuhara et al. 1997; Pussard et al. 2007)
Ivermectin	45	26.4–59.2 (Geyer et al. 2009; Kiki-Mvouaka et al. 2010; Schinkel et al. 1994)
Indinavir	4.5	9.4–21.3 (Chu et al. 2012; Kim et al. 1998)
Vincristine	4.3	1.4 (Wang et al. 2010)
Paclitaxel	1.0	2.0–7.9 (Gallo et al. 2003; Kemper et al. 2003)
Olanzapine	3.9	2.7 (Wang et al. 2004)
Citalopram	1.0	2.0–3.5 (Bundgaard et al. 2012; Uhr and Grauer 2003)

Simulations are performed in an “average” adult (35 y) or pediatric (1 mnd) individual

## Discussion

Transport across the blood–brain barrier was modelled for drugs that enter the brain via passive diffusion only and for typical P-gp drug substrates. Incorporation of active transport in the model improved the prediction of human adult and paediatric CSF drug concentrations for Pgp substrates, although this was not necessary for drugs entering brain via passive diffusion (Figs. 3, 4 and 5). The effect of Pgp activity on brain drug exposure tended to be lower according to the human simulation as compared to mouse and rat Pgp-knockout/-inhibition studies (Table 2).

Interspecies variability in the influence of Pgp on brain drug concentrations can be explained by differences in transporter expression, as well as transporter activity. Compared to human, Pgp expression in mouse is 2.3-fold higher and in rat about fourfold higher (Al Feteisi et al. 2018; Uchida et al. 2011). In vitro studies also point towards a variable activity and different substrate affinity of human Pgp compared with the mouse orthologue. However, this needs further investigation, as variable transporter activity between species has not been normalized for expression differences in the in vitro experimental systems (Schinkel et al. 1995; Xia et al. 2006; Yamazaki et al. 2001).

The verapamil model simulations reported in this study are in line with clinical human ^11^C-verapamil PET studies, where (near) complete inhibition of Pgp by tariquidar was achieved. Brain disposition increased by 273%, which is close to the ~ 2.3-fold difference predicted here, but far below the up to 10 × higher Kp ratios reported in rats and mice, which is can be explained by differences in expression or transporter affinity (Table 2) (Bauer et al. 2015; Zolnerciks et al. 2011). This is also in line with higher brain-to-plasma Kp values reported in rat compared to human and monkey for the Pgp substrates ^11^C-GR205171 and ^18^F-altanserin, even when corrected for differences in plasma protein binding and clearance (Syvänen et al. 2009). Therefore, species differences in transporter activity tend towards a lower Pgp activity in human BBB in accordance with our results, however, this can be substrate dependent as shown for vincristine (Syvänen et al. 2009).

Differences in brain exposure between animals and humans could have consequences for toxicity testing and drug development. Although species differences are often considered in toxicity testing by taking into account an interspecies safety factor of 10, of which a factor 4 is used specifically for inter-species differences in toxicokinetics, differences in blood–brain barrier drug disposition already can outrange this value when evaluating Pgp transporter substrates (Dankovic et al. 2015). Pgp activity has also been a topic of interest in drug development for CNS diseases, as substrates are less likely to reach the target site (Mahar Doan et al. 2002). The findings in this study indicate that while quantification of Pgp activity in rodent species appears insightful in an early stage of the drug development process, it might result in an underestimation of human brain exposure to Pgp substrates when no corrections between species are considered, which will be even more pronounced for children due to a lower Pgp expression (Verscheijden et al. 2020b). In future studies, a human PBPK model approach could be used in parallel to animal studies to evaluate brain disposition for Pgp substrates. In addition, age-dependent changes in transporter expression could be taken into account. Furthermore, rat and mouse PBPK models developed in parallel could facilitate the translational step from rodent to human PK, as this provides an opportunity to elucidate differences in transporter activity using species-specific in vitro data.

The study described here has several limitations. An important aspect to consider when using in vitro and ex vivo results to quantify *in* vivo effects, is the quality and variability of available transporter activity and abundance data. In this study, optimization of transporter abundance was required using CSF measurements before acceptable predictions could be made for digoxin, verapamil and quinidine (Li et al. 2017). Inter-laboratory differences in the proteomic measurements of transporter abundance can be large, which require standardization of procedures (Harwood et al. 2016). In addition, quantitative proteomics would ideally be performed on the same cells used in the in vitro transport experiments to minimize the effect of culture conditions. Currently, verification of model-predicted transporter activity is therefore required. A second limitation is that predicted brain mass exposures could not be validated with measured data, as they were not available for the compounds studied. Drug concentrations in CSF will not be the same as in ECF, but are strongly linked, *e.g.* due to the flow of drugs from brain mass to the cranial CSF. This means that accurate predictions in the CSF provide indirect information about the accuracy of the simulated drug concentrations in brain. In addition, the human brain model proved suitable for predicting brain parenchyma concentrations in previous studies, as was shown for morphine and AZD1775, but these compounds are less clear BBB Pgp substrates and therefore not used here (Li et al. 2017; Verscheijden et al. 2021). Predicted Kp ratios also correlated well with human clinical ^11^C-verapamil PET values, as discussed above (Bauer et al. 2015; Li et al. 2017). Third, Kp values in rodents are not always derived at plasma concentrations comparable to the human clinical studies. This is the case for olanzapine and indinavir, where plasma concentrations in rat where about tenfold higher and fivefold lower than in human plasma, respectively. Drug plasma concentrations are substantially below Km values for olanzapine, but around Km for indinavir, which might result in saturation of transporters (Boulton et al. 2002; Lee et al. 1998). For the other Pgp substrates, Kp values were determined at similar plasma concentrations in humans and rodents. Last, whereas all transporter substrates considered are transported by Pgp, other efflux transporters might contribute. This could result in an overestimation of the predicted Pgp effect in human as compared to mouse Pgp knockout and inhibition studies. The observed trend indicating more Pgp activity in rodents compared to human studies would in this case be even more pronounced. Uptake transporter activity potentially differs between species and could become important when compounds would be substrates such as paclitaxel which is described to be a substrate for hepatic OATP2B1 (Tanino et al. 2009). However, species differences in uptake transporter activity are unknown and for most Pgp substrates reported here, no BBB uptake transporter activity has been described.

The simulations in this study indicate that a PBPK model-based approach can be used to quantify human adult CSF drug concentrations without clinical data, but only in case when robust in vitro data and scaling factors would be available. In addition, age-appropriate inclusion of Pgp can result in an improved prediction in CSF in children. BBB transport in PBPK models has often been scaled using clinically measured PK data in case no in vitro transport data were available (Gaohua et al. 2016; Li et al. 2017). The advantage of including kinetic parameters determined in vitro is, however, that model predictions are not necessarily dependent on clinically measured data, which could be a valuable strategy in the future in case no brain ECF or CSF samples are available.

In conclusion, a PBPK model was developed to predict human brain disposition for various Pgp substrates. Model simulations were verified with measurements in cerebrospinal fluid. The influence of BBB Pgp activity appeared generally more pronounced in knockout mice and rats compared to the human PBPK model. This indicates that assessment of the central nervous system activity of Pgp substrates in rodents might result in an underestimation of their human efficacy and toxicity.

## Supplementary Information

Below is the link to the electronic supplementary material.

Supplementary file1 (PDF 185 kb)

## Data Availability

All data generated or analyzed during this study are included in this published article, its supplementary information files, and references.
