# Supplementary material for: Differences in P-glycoprotein activity in human and rodent blood–brain barrier assessed by mechanistic modelling
Source: Arch Toxicol. 2021 Jul 15;95(9):3015–29. doi: 10.1007/s00204-021-03115-y (PMC8380243; doi:10.1007/s00204-021-03115-y)
Supplement: Supplementary file 1 — Supplementary file1 (PDF 185 kb) [file 204_2021_3115_MOESM1_ESM.pdf]

Quetiapine

Parameter	Value	Reference
Molecular weight	384 g/mol	(1)
LogP	2.86	(1)
Pka	3.45, 6.93 (diprotic base)	(1)
Fa	1	(1)
Ka	1.77 h-1	(1)
Blood-plasma ratio	1.26	(1)
Fraction unbound plasma	0.17	(1)
Kp-scalar	3.5	Optimized
CL	101.5 L/h	(1)
CL-scalar	1	Optimized
Papp *in vitro*	$33*10^{-6}$ cm/s	(2)
Fraction unbound brain mass	0.025	(3)

Oxycodone

Parameter	Value	Reference
Molecular weight	315.4 g/mol	pubchem
LogP	1.2	pubchem
Pka	8.53 (base)	pubchem
Blood-plasma ratio	1.3	(4)
Fraction unbound plasma	0.59	(5)
Kp-scalar	0.4	optimized
CL	49.2 L/h	(6)
CL-scalar	0.8	optimized
Papp *in vitro*	$16.9*10^{-6}$ cm/s	(7-9)
Fraction unbound brain mass	0.45	(10) (1/Vu,brain)

Mirtazapine

Parameter	Value	References
Molecular weight	265.4	Pubchem
LogP	3.3	pubchem
Pka	7.7 (basic)	Pubchem
Fa	0.5	(11)
Ka	1	-
Blood-plasma ratio	1	-
Fraction unbound plasma	0.15	(11)
Kp-scalar	1	optimized
CL	31.2	(11)
CL-scalar	1	optimized
Papp *in vitro*	$28.4*10^{-6}$ cm/s	(2, 12-14)
Fraction unbound brain mass	0.08	(15)

Etoricoxib

Parameter	Value	References
Molecular weight	358.8	pubchem
LogP	3.3	Pubchem
Pka	4.96	drugbank
Fa	0.83	(16)
Ka	1	-
Blood-plasma ratio	1	-
Fraction unbound plasma	0.08	(16)
Kp-scalar	0.25	optimized
CL	3.42	(16)
CL-scalar	1	optimized
Papp *in vitro*	$52.3*10^{-6}$ cm/s	(17)
Fraction unbound brain mass	0.15	(18)

Dexketoprofen

Parameter	Value	References
Molecular weight	254.3	Pubchem
LogP	3.1	Pubchem
Pka	3.88 (acid)	Drugbank
Blood-plasma ratio	0.56	(19)
Fraction unbound plasma	0.017	(19)
Kp-scalar	1	optimized
CL	5.19 L/h	(19)
CL-scalar	1.9	optimized
Papp *in vitro*	$44.3*10^{-6}$ cm/s	(20-22)
Fraction unbound brain mass	0.93	Simcyp predicted value

Lacosamide

Parameter	Value	References
Molecular weight	250.3	Pubchem
LogP	0.3	Pubchem
Pka	neutral	drugbank
Fa	1	(23)
Ka	2.45	(24)
Blood-plasma ratio	1	-
Fraction unbound plasma	1	(25)
Kp-scalar	0.5	optimized
CL	2.13	(26)
CL-scalar	0.8	optimized
Papp *in vitro*	$18.8*10^{-6}$	(27)
Fraction unbound brain mass	0.9	(28)

Citalopram

Parameter	Value	References
Molecular weight	324.4	pubchem

LogP	3.2	Pubchem
Pka	9.78 (base)	Pubchem
Fa	1	(29)
Ka	1	-
Blood-plasma ratio	1	-
Fraction unbound plasma	0.2	(30)
Kp-scalar	1	optimized
CL	24.6	(31)
CL-scalar	0.33	optimized
Papp *in vitro*	$50.1*10^{-6}$ cm/s	(2, 12, 32, 33)
CL pgp efflux	0 L/h	Optimized
Fraction unbound brain mass	0.031	(34)

Digoxin

**Parameter**	**Value**	**References**
Molecular weight	780.9	(35)
LogP	1.26	(35)
Pka	13.5 (acid)	Pubchem
Fa	0.59	(35)
Ka	0.82 h-1	(35)
Blood-plasma ratio	1.07	(35)
Fraction unbound plasma	0.71	(35)
Kp-scalar	1	Optimized
CL	13.46 L/h	(35)
CL-scalar	0.67	Optimized
Papp *in vitro*	$10.5*10^{-6}$ cm/s	(33, 36-38)
Pgp Vmax	434 pmol/min/cm2	(35)
Pgp Km	177 uM	(35)
Fraction unbound brain mass	0.104	(39)

Verapamil

**Parameter**	**Value**	**References**
Molecular weight	454.60 g/mol	(40)
LogP	3.81	(40)
Pka	8.92	(40)
Fa	0.99	Simcyp prediction, (40)
Ka	2.79 h-1	Simcyp prediction, (40)
Blood-plasma ratio	0.71	(40)
Fraction unbound plasma	0.091	(40)
Kp-scalar	2.78	(40)
CL	55.37 L/h	(40)
CL-scalar	1.26	Optimized
Papp *in vitro*	$23.63*10^{-6}$ cm/s	(41-43)
Pgp Vmax	18.4 pmol/min/cm2	(40)
Pgp Km	3.1 uM	(40)
Fraction unbound brain mass	0.76	Simcyp prediction

Quinidine

Parameter	Value	References
Molecular weight	324.4 g/mol	Simcyp compound file
LogP	2.81	Simcyp compound file
Pka	4.2, 8.8 (diprotic base)	Simcyp compound file
Fa	1	Simcyp compound file
Ka	3 h-1	Simcyp compound file
Blood-plasma ratio	0.82	Simcyp compound file
Fraction unbound plasma	0.202	Simcyp compound file
Kp-scalar	1	Optimized
CL	18.2 L/h	(44)
CL-scalar	1.65	Optimized
Papp *in vitro*	$19.2*10^{-6}$ cm/s	(41, 45, 46)
Pgp Vmax	12.0 pmol/min/cm2	(36)
Pgp Km	2.20 uM	(36)
Fraction unbound brain mass	0.247	Simcyp predicted value

Vincristine

Parameter	Value	References
Molecular weight	824.96 g/mol	(47)
LogP	2.82	(47)
Pka	5, 7.4 (diprotic base)	(47)
Blood-plasma ratio	2.15	Simcyp predicted value
Fraction unbound plasma	0.51	(47)
Kp-scalar	0.4	Optimized
CL	8.5 L/h	(48)
CL-scalar	1	Optimized
Papp *in vitro*	$2.88*10^{-6}$ cm/s	(49)
CL Pgp efflux	8 L/h	optimized
Fraction unbound brain mass	0.0334	Simcyp predicted value

Indinavir

Parameter	Value	References
Molecular weight	613.8 g/mol	Pubchem
LogP	2.9	
Pka	7.4	Drugbank
Fa	0.65	
Ka	1 h-1	-
Blood-plasma ratio	1.2	(50)
Fraction unbound plasma	0.559	(51)
Kp-scalar	0.1	Optimized
CL	47.9 L/h	(52)
CL-scalar	1	Optimized
Papp *in vitro*	$53.2*10^{-6}$ cm/s	(53)
Cl Pgp efflux	150 L/h	Optimized
Fraction unbound brain mass	0.145	(54)

Ivermectin

Parameter	Value	References
Molecular weight	875.1 g/mol	Pubchem
LogP	4.1	Pubchem
Pka	Neutral	Drugbank
Blood-plasma ratio	2.7	(55)
Fraction unbound plasma	0.068	(56)
Kp-scalar	0.1	Optimized
CL	7.67	(57)
CL-scalar	0.63	Optimized
Papp *in vitro*	$9.1*10^{-6}$ cm/s	(58)
CL pgp efflux	320 L/h	Optimized
Fraction unbound brain mass	0.00009	(59)

Docetaxel

Parameter	Value	References
Molecular weight	809.7 g/mol	Pubchem
LogP	2.4	Puchem
Pka	neutral	Drugbank
Blood-plasma ratio	0.71	(60)
Fraction unbound plasma	0.07	(61)
Kp-scalar	50	Optimized
CL	80.8 L/h	(61)
CL-scalar	0.56	Optimized
Papp *in vitro*	$0.155*10^{-6}$ cm/s	(62)
CL Pgp efflux	3 L/h	Optimized
Fraction unbound brain mass	0.0262	(63)

Paclitaxel

Parameter	Value	References
Molecular weight	853.9 g/mol	Pubchem
LogP	3	Pubchem
Pka	10.4	Drugbank
Blood-plasma ratio	1	-
Fraction unbound plasma	0.039	(64)
Kp-scalar	2	Optimized
CL	12*Body surface area, L/h	(65)
CL-scalar	1.2	Optimized
Papp *in vitro*	$26.9*10^{-6}$ cm/s	(66)
CL Pgp efflux	0 L/h	Optimized
Fraction unbound brain mass	0.00488	(66)

Olanzapine

Parameter	Value	References
Molecular weight	312.4	Pubchem
LogP	3	Pubchem
Pka	7.2 (basic)	(67)
Fa	1	(67)
Ka	1.36 h-1	(67)
Blood-plasma ratio	0.73	(67)
Fraction unbound plasma	0.327	(67)
Kp-scalar	2	Optimized
CL	26 L/h	(68)
CL-scalar	0.46	Optimized
Papp *in vitro*	$10.7*10^{-6}$ cm/s	(2, 15)
CL Pgp efflux	25 L/h	Optimized
Fraction unbound brain mass	0.034	(15)

References

1. Johnson TN, Zhou D, Bui KH. Development of physiologically based pharmacokinetic model to evaluate the relative systemic exposure to quetiapine after administration of IR and XR formulations to adults, children and adolescents. Biopharm Drug Dispos. 2014;35(6):341-52.
2. Di L, Kerns EH, Bezar IF, Petusky SL, Huang Y. Comparison of blood-brain barrier permeability assays: in situ brain perfusion, MDR1-MDCKII and PAMPA-BBB. J Pharm Sci. 2009;98(6):1980-91.
3. Watson J, Wright S, Lucas A, Clarke KL, Viggers J, Cheetham S, et al. Receptor occupancy and brain free fraction. Drug Metab Dispos. 2009;37(4):753-60.
4. Moore C, Kelley-Baker T, Lacey J. Interpretation of oxycodone concentrations in oral fluid. J Opioid Manag. 2012;8(3):161-6.
5. Korjamo T, Tolonen A, Ranta VP, Turpeinen M, Kokki H. Metabolism of oxycodone in human hepatocytes from different age groups and prediction of hepatic plasma clearance. Front Pharmacol. 2011;2:87.
6. Kinnunen M, Piirainen P, Kokki H, Lammi P, Kokki M. Updated Clinical Pharmacokinetics and Pharmacodynamics of Oxycodone. Clin Pharmacokinet. 2019;58(6):705-25.
7. Hassan HE, Myers AL, Lee IJ, Coop A, Eddington ND. Oxycodone induces overexpression of P-glycoprotein (ABCB1) and affects paclitaxel's tissue distribution in Sprague Dawley rats. J Pharm Sci. 2007;96(9):2494-506.
8. Volpe DA, Asafu-Adjaye EB, Ellison CD, Doddapaneni S, Uppoor RS, Khan MA. Effect of ethanol on opioid drug permeability through caco-2 cell monolayers. Aaps j. 2008;10(2):360-2.
9. Ball K, Bouzom F, Scherrmann JM, Walther B, Declèves X. Development of a physiologically based pharmacokinetic model for the rat central nervous system and determination of an in vitro-in vivo scaling methodology for the blood-brain barrier permeability of two transporter substrates, morphine and oxycodone. J Pharm Sci. 2012;101(11):4277-92.
10. Hammarlund-Udenaes M, Fridén M, Syvänen S, Gupta A. On the rate and extent of drug delivery to the brain. Pharm Res. 2008;25(8):1737-50.
11. Timmer CJ, Sitsen JM, Delbressine LP. Clinical pharmacokinetics of mirtazapine. Clin Pharmacokinet. 2000;38(6):461-74.
12. Zheng Y, Chen X, Benet LZ. Reliability of In Vitro and In Vivo Methods for Predicting the Effect of P-Glycoprotein on the Delivery of Antidepressants to the Brain. Clin Pharmacokinet. 2016;55(2):143-67.

13. O'Brien FE, Clarke G, Dinan TG, Cryan JF, Griffin BT. Human P-glycoprotein differentially affects antidepressant drug transport: relevance to blood-brain barrier permeability. Int J Neuropsychopharmacol. 2013;16(10):2259-72.
14. Yau E, Petersson C, Dolgos H, Peters SA. A comparative evaluation of models to predict human intestinal metabolism from nonclinical data. Biopharm Drug Dispos. 2017;38(3):163-86.
15. Heymans M, Sevin E, Gosselet F, Lundquist S, Culot M. Mimicking brain tissue binding in an in vitro model of the blood-brain barrier illustrates differences between in vitro and in vivo methods for assessing the rate of brain penetration. Eur J Pharm Biopharm. 2018;127:453-61.
16. Takemoto JK, Reynolds JK, Remsberg CM, Vega-Villa KR, Davies NM. Clinical pharmacokinetic and pharmacodynamic profile of etoricoxib. Clin Pharmacokinet. 2008;47(11):703-20.
17. Mitra A, Kesisoglou F, Dogterom P. Application of absorption modeling to predict bioequivalence outcome of two batches of etoricoxib tablets. AAPS PharmSciTech. 2015;16(1):76-84.
18. Summerfield SG, Stevens AJ, Cutler L, del Carmen Osuna M, Hammond B, Tang SP, et al. Improving the in vitro prediction of in vivo central nervous system penetration: integrating permeability, P-glycoprotein efflux, and free fractions in blood and brain. J Pharmacol Exp Ther. 2006;316(3):1282-90.
19. Ye M, Nagar S, Korzekwa K. A physiologically based pharmacokinetic model to predict the pharmacokinetics of highly protein-bound drugs and the impact of errors in plasma protein binding. Biopharm Drug Dispos. 2016;37(3):123-41.
20. Sun D, Lennernas H, Welage LS, Barnett JL, Landowski CP, Foster D, et al. Comparison of human duodenum and Caco-2 gene expression profiles for 12,000 gene sequences tags and correlation with permeability of 26 drugs. Pharm Res. 2002;19(10):1400-16.
21. Larregieu CA, Benet LZ. Drug discovery and regulatory considerations for improving in silico and in vitro predictions that use Caco-2 as a surrogate for human intestinal permeability measurements. Aaps j. 2013;15(2):483-97.
22. Hilgendorf C, Spahn-Langguth H, Regårdh CG, Lipka E, Amidon GL, Langguth P. Caco-2 versus Caco-2/HT29-MTX co-cultured cell lines: permeabilities via diffusion, inside- and outside-directed carrier-mediated transport. J Pharm Sci. 2000;89(1):63-75.
23. Cawello W, Boekens H, Bonn R. Absorption, disposition, metabolic fate and elimination of the anti-epileptic drug lacosamide in humans: mass balance following intravenous and oral administration. Eur J Drug Metab Pharmacokinet. 2012;37(4):241-8.
24. Winkler J, Schoemaker R, Stockis A. Population Pharmacokinetics of Adjunctive Lacosamide in Pediatric Patients With Epilepsy. J Clin Pharmacol. 2019;59(4):541-7.
25. Johannessen Landmark C, Patsalos PN. Drug interactions involving the new second- and third-generation antiepileptic drugs. Expert Rev Neurother. 2010;10(1):119-40.
26. Cawello W, Fuhr U, Hering U, Maatouk H, Halabi A. Impact of impaired renal function on the pharmacokinetics of the antiepileptic drug lacosamide. Clin Pharmacokinet. 2013;52(10):897-906.
27. Zhang C, Chanteux H, Zuo Z, Kwan P, Baum L. Potential role for human P-glycoprotein in the transport of lacosamide. Epilepsia. 2013;54(7):1154-60.
28. Gáll Z, Vancea S. Distribution of lacosamide in the rat brain assessed by in vitro slice technique. Arch Pharm Res. 2018;41(1):79-86.
29. van Harten J. Clinical pharmacokinetics of selective serotonin reuptake inhibitors. Clin Pharmacokinet. 1993;24(3):203-20.
30. Paulzen M, Lammertz SE, Gründer G, Veselinovic T, Hiemke C, Tauber SC. Measuring citalopram in blood and cerebrospinal fluid: revealing a distribution pattern that differs from other antidepressants. Int Clin Psychopharmacol. 2016;31(3):119-26.
31. Kragh-Sørensen P, Overø KF, Petersen OL, Jensen K, Parnas W. The kinetics of citalopram: single and multiple dose studies in man. Acta Pharmacol Toxicol (Copenh). 1981;48(1):53-60.
32. Rochat B, Baumann P, Audus KL. Transport mechanisms for the antidepressant citalopram in brain microvessel endothelium. Brain Res. 1999;831(1-2):229-36.

33. Sevin E, Dehouck L, Fabulas-da Costa A, Cecchelli R, Dehouck MP, Lundquist S, et al. Accelerated Caco-2 cell permeability model for drug discovery. J Pharmacol Toxicol Methods. 2013;68(3):334-9.
34. Bundgaard C, Jensen CJ, Garmer M. Species comparison of in vivo P-glycoprotein-mediated brain efflux using mdr1a-deficient rats and mice. Drug Metab Dispos. 2012;40(3):461-6.
35. Neuhoff S, Yeo KR, Barter Z, Jamei M, Turner DB, Rostami-Hodjegan A. Application of permeability-limited physiologically-based pharmacokinetic models: part I-digoxin pharmacokinetics incorporating P-glycoprotein-mediated efflux. J Pharm Sci. 2013;102(9):3145-60.
36. Troutman MD, Thakker DR. Efflux ratio cannot assess P-glycoprotein-mediated attenuation of absorptive transport: asymmetric effect of P-glycoprotein on absorptive and secretory transport across Caco-2 cell monolayers. Pharm Res. 2003;20(8):1200-9.
37. Djuv A, Nilsen OG. Caco-2 cell methodology and inhibition of the P-glycoprotein transport of digoxin by Aloe vera juice. Phytother Res. 2008;22(12):1623-8.
38. Elsby R, Surry DD, Smith VN, Gray AJ. Validation and application of Caco-2 assays for the in vitro evaluation of development candidate drugs as substrates or inhibitors of P-glycoprotein to support regulatory submissions. Xenobiotica. 2008;38(7-8):1140-64.
39. Mihajlica N, Betsholtz C, Hammarlund-Udenaes M. Pharmacokinetics of pericyte involvement in small-molecular drug transport across the blood-brain barrier. Eur J Pharm Sci. 2018;122:77-84.
40. Neuhoff S, Yeo KR, Barter Z, Jamei M, Turner DB, Rostami-Hodjegan A. Application of permeability-limited physiologically-based pharmacokinetic models: part II - prediction of P-glycoprotein mediated drug-drug interactions with digoxin. J Pharm Sci. 2013;102(9):3161-73.
41. Shirasaka Y, Masaoka Y, Kataoka M, Sakuma S, Yamashita S. Scaling of in vitro membrane permeability to predict P-glycoprotein-mediated drug absorption in vivo. Drug Metab Dispos. 2008;36(5):916-22.
42. Li C, Liu T, Cui X, Uss AS, Cheng KC. Development of in vitro pharmacokinetic screens using Caco-2, human hepatocyte, and Caco-2/human hepatocyte hybrid systems for the prediction of oral bioavailability in humans. J Biomol Screen. 2007;12(8):1084-91.
43. Feng B, Mills JB, Davidson RE, Mireles RJ, Janiszewski JS, Troutman MD, et al. In vitro P-glycoprotein assays to predict the in vivo interactions of P-glycoprotein with drugs in the central nervous system. Drug Metab Dispos. 2008;36(2):268-75.
44. Ochs HR, Greenblatt DJ, Woo E, Smith TW. Reduced quinidine clearance in elderly persons. Am J Cardiol. 1978;42(3):481-5.
45. Furubayashi T, Inoue D, Nishiyama N, Tanaka A, Yutani R, Kimura S, et al. Comparison of Various Cell Lines and Three-Dimensional Mucociliary Tissue Model Systems to Estimate Drug Permeability Using an In Vitro Transport Study to Predict Nasal Drug Absorption in Rats. Pharmaceutics. 2020;12(1).
46. Sziráki I, Erdo F, Beéry E, Molnár PM, Fazakas C, Wilhelm I, et al. Quinidine as an ABCB1 probe for testing drug interactions at the blood-brain barrier: an in vitro in vivo correlation study. J Biomol Screen. 2011;16(8):886-94.
47. Lee CM, Zane NR, Veal G, Thakker DR. Physiologically Based Pharmacokinetic Models for Adults and Children Reveal a Role of Intracellular Tubulin Binding in Vincristine Disposition. CPT Pharmacometrics Syst Pharmacol. 2019;8(10):759-68.
48. Sethi VS, Jackson DV, Jr., White DR, Richards F, 2nd, Stuart JJ, Muss HB, et al. Pharmacokinetics of vincristine sulfate in adult cancer patients. Cancer Res. 1981;41(9 Pt 1):3551-5.
49. Pade D, Jamei M, Rostami-Hodjegan A, Turner DB. Application of the MechPeff model to predict passive effective intestinal permeability in the different regions of the rodent small intestine and colon. Biopharm Drug Dispos. 2017;38(2):94-114.
50. De Bruyn T, Augustijns PF, Annaert PP. Hepatic Clearance Prediction of Nine Human Immunodeficiency Virus Protease Inhibitors in Rat. J Pharm Sci. 2016;105(2):846-53.
51. Haas DW, Johnson B, Nicotera J, Bailey VL, Harris VL, Bowles FB, et al. Effects of ritonavir on indinavir pharmacokinetics in cerebrospinal fluid and plasma. Antimicrob Agents Chemother. 2003;47(7):2131-7.

52. DiCenzo R, Forrest A, Fischl MA, Collier A, Feinberg J, Ribaudo H, et al. Pharmacokinetics of indinavir and nelfinavir in treatment-naive, human immunodeficiency virus-infected subjects. Antimicrob Agents Chemother. 2004;48(3):918-23.
53. Kapitza SB, Michel BR, van Hoogevest P, Leigh ML, Imanidis G. Absorption of poorly water soluble drugs subject to apical efflux using phospholipids as solubilizers in the Caco-2 cell model. Eur J Pharm Biopharm. 2007;66(1):146-58.
54. Culot M, Fabulas-da Costa A, Sevin E, Szorath E, Martinsson S, Renftel M, et al. A simple method for assessing free brain/free plasma ratios using an in vitro model of the blood brain barrier. PLoS One. 2013;8(12):e80634.
55. Schmith VD, Zhou JJ, Lohmer LRL. The Approved Dose of Ivermectin Alone is not the Ideal Dose for the Treatment of COVID-19. Clin Pharmacol Ther. 2020;108(4):762-5.
56. Klotz U, Ogbuokiri JE, Okonkwo PO. Ivermectin binds avidly to plasma proteins. Eur J Clin Pharmacol. 1990;39(6):607-8.
57. Duthaler U, Suenderhauf C, Karlsson MO, Hussner J, Meyer Zu Schwabedissen H, Krähenbühl S, et al. Population pharmacokinetics of oral ivermectin in venous plasma and dried blood spots in healthy volunteers. Br J Clin Pharmacol. 2019;85(3):626-33.
58. Kigen G, Edwards G. Drug-transporter mediated interactions between anthelminthic and antiretroviral drugs across the Caco-2 cell monolayers. BMC Pharmacol Toxicol. 2017;18(1):20.
59. Kalvass JC, Maurer TS, Pollack GM. Use of plasma and brain unbound fractions to assess the extent of brain distribution of 34 drugs: comparison of unbound concentration ratios to in vivo p-glycoprotein efflux ratios. Drug Metab Dispos. 2007;35(4):660-6.
60. Dumez H, Guetens G, De Boeck G, Highley MS, de Bruijn EA, van Oosterom AT, et al. In vitro partition of docetaxel and gemcitabine in human volunteer blood: the influence of concentration and gender. Anticancer Drugs. 2005;16(8):885-91.
61. ten Tije AJ, Loos WJ, Zhao M, Baker SD, Enting RH, van der Meulen H, et al. Limited cerebrospinal fluid penetration of docetaxel. Anticancer Drugs. 2004;15(7):715-8.
62. Liu H, Tu L, Zhou Y, Dang Z, Wang L, Du J, et al. Improved Bioavailability and Antitumor Effect of Docetaxel by TPGS Modified Proniosomes: In Vitro and In Vivo Evaluations. Sci Rep. 2017;7:43372.
63. Wang Q, Ren T, Zhao J, Wong CH, Chan HYE, Zuo Z. Exclusion of unsuitable CNS drug candidates based on their physicochemical properties and unbound fractions in biomatrices for brain microdialysis investigations. J Pharm Biomed Anal. 2020;178:112946.
64. van den Bongard HJ, Kemper EM, van Tellingen O, Rosing H, Mathôt RA, Schellens JH, et al. Development and validation of a method to determine the unbound paclitaxel fraction in human plasma. Anal Biochem. 2004;324(1):11-5.
65. Stage TB, Bergmann TK, Kroetz DL. Clinical Pharmacokinetics of Paclitaxel Monotherapy: An Updated Literature Review. Clin Pharmacokinet. 2018;57(1):7-19.
66. Uchida Y, Ohtsuki S, Kamiie J, Terasaki T. Blood-brain barrier (BBB) pharmacoproteomics: reconstruction of in vivo brain distribution of 11 P-glycoprotein substrates based on the BBB transporter protein concentration, in vitro intrinsic transport activity, and unbound fraction in plasma and brain in mice. J Pharmacol Exp Ther. 2011;339(2):579-88.
67. Reddy VP, Jones BC, Colclough N, Srivastava A, Wilson J, Li D. An Investigation into the Prediction of the Plasma Concentration-Time Profile and Its Interindividual Variability for a Range of Flavin-Containing Monooxygenase Substrates Using a Physiologically Based Pharmacokinetic Modeling Approach. Drug Metab Dispos. 2018;46(9):1259-67.
68. Callaghan JT, Bergstrom RF, Ptak LR, Beasley CM. Olanzapine. Pharmacokinetic and pharmacodynamic profile. Clin Pharmacokinet. 1999;37(3):177-93.
